# Supplementary material for: Biological Graft as an Innovative Biomaterial for Complex Skin Wound Treatment in Dogs: A Preliminary Report
Source: Materials (Basel). 2022 Sep 1;15(17):6027. doi: 10.3390/ma15176027 (PMC9456771; doi:10.3390/ma15176027)
Supplement: Supplementary file 1 [file materials-15-06027-s001.zip › materials-1832817-supplementary.pdf]

# Biological Graft as an Innovative Biomaterial for Complex Skin Wound Treatment in Dogs: A Preliminary Report

**Adriano Jaskonis Dall'Olio [1], Gustavo de Sá Schiavo Matias [1], Ana Claudia Oliveira Carreira [1], Hianka Jasmyne Costa de Carvalho [1], Thais van den Broek Campanelli [1], Thamires Santos da Silva [1], Mônica Duarte da Silva [1], Ana Lúcia Abreu-Silva [2] and Maria Angélica Miglino [1,*]**

[1] Surgery Department, School of Veterinary Medicine and Animal Science, University of São Paulo, São Paulo 05508-270, Brazil
[2] Department of Veterinary Pathology, State University of Maranhão, Maranhão 65055-150, Brazil
* Correspondence: miglino@usp.br; Tel.: +55-(11)-3091-7690

## Supplementary data

**Supplementary Table S1.** Electrocardiogram, hematological and biochemical results of the dogs before scaffolds grafting.

| DOGS | RBC (millions/mm³) | HGB (g/dl) | HCT (%) | TP (g/dl) | PLT (1,000/mm³) | LEU (1,000/mm³) | CR (mg/dl) | BUN (mg/dl) | ALT (U/L) | ALP (U/L) | GLU (mg/dl) | ECG |
|---|---|---|---|---|---|---|---|---|---|---|---|---|
| Dog 1 (Group 1) | 6.5 | 14.7 | 47 | 7.2 | 260 | 8,700 | 0.8 | 36.5 | 47.1 | 108 | 90 | No alterations |
| Dog 2 (Group 1) | 6.7 | 15.9 | 50 | 6.6 | 370 | 10,300 | 1.1 | 20.8 | 26.8 | 74 | 86 | No alterations |
| Dog 3 (Group 1) | 6.1 | 14.2 | 45 | 8 | 500 | 19,500 | 0.5 | 15.7 | 52.3 | 116 | 67 | No alterations |
| Dog 4 (Group 2) | 6.5 | 13.5 | 41 | 7.4 | 670 | 10,800 | 1.1 | 33.5 | 36.6 | 539 | 77 | No alterations |
| Dog 5 (Group 2) | 7.1 | 15.6 | 53 | 7.2 | 139 | 6,800 | 1.4 | 31.7 | 54.1 | 58 | 80 | No alterations |

| | | | | | | | | | | | | |
|---|---|---|---|---|---|---|---|---|---|---|---|---|
| Dog 6 (Group 2) | 6.1 | 14.3 | 50 | 7 | 190 | 7,200 | 1.3 | 35.4 | 56.2 | 63 | 75 | No alterations |
| Dog 7 (Group 3) | 5.7 | 12.4 | 38 | 6.6 | 220 | 9,300 | 1.2 | 58 | 73.1 | 126 | 72 | No alterations |
| Dog 8 (Group 3) | 6.3 | 13.8 | 45 | 7.2 | 460 | 10,600 | 1.1 | 54 | 70 | 115 | 68 | No alterations |

RBC: Red blood cells; HGB: Hemoglobin. HCT: Hematocrit; TP: Total protein; PLT: Platelet Count; LEU: Leukocytes; CRE: Creatinine; BUN: Blood Urea Nitrogen; ALT: Alanine Aminotransferase; ALP: Alkaline Phosphatase; Glu: Glucose; ECG: Electrocardiogram.

**Supplementary Table S2**. Results indicating the patient's anamnesis and the macroscopic characteristics of the wounds before and after scaffolds grafting in dogs from group I.

| Dogs | Anamnesis | Wound clinical analysis (Day 0) | Wound evolution post-grafting | Complete Healing |
|---|---|---|---|---|
| **Dog 1 (Group I)** | Presence of ulcerative lesion starting 6 months ago. Application of topical treatment, but with local complication and recurrence. | Alopecic lesion in Left metatarsal region, firm and crusted, with moderate exudative secretion and edematous and ulcerated tissue. | After 48 hours of grafting, a process of integration of the scaffold to the wound, reduction of edema and some whitish regions in the center with non-adherence of the scaffold to the tissue was observed. At 10 days, a reduction in the size of the wound and retraction of the edges with approximation was observed, and at 20 days there was re-epithelialization and initial dog fur formation. | Closure and complete healing with formation of skin attachments at 31 days. |

| | | | | |
|---|---|---|---|---|
| **Dog 2 (Group I)** | Laceration in the right lateral thoracic region due to a capybara bite. Clinically, an open, deep and contaminated wound with purulent exudate was observed. | Focal reddish ulcerated lesion in Right lateral thoracic (costal) region, with loss of superficial and deep dermis and exposure of the muscular layer. | 48 hours after grafting, the scaffold began to integrate with the injured tissue, demonstrating greater adherence between the scaffold and tissue in some points. At 10 days, it was verified that the wound had regular edges and surface. | Established wound 40 days after grafting. |
| **Dog 3 (Group I)** | Progressive increase in volume at the right thoracic limb for at least 2 years, with the presence of an ulcerated nodule. | Adhered neoformation was observed in the Right metacarpal region with an intensely ulcerated surface and necrotic areas with exudate presence. | After 48 hours, partial graft presence, irregular surface and edema were observed. After 10 days, peripheral scar tissue formation with a whitish color. After 20 days, there was an approximation of the wound edges and a reduction in its diameter. | Wound established 45 days after grafting. Total wound healing. |

**Supplementary Table S3**. Results indicating the patient's anamnesis and the macroscopic characteristics of the wounds before and after scaffolds grafting in dogs from group II.

| Dogs | Historic | Wound Clinical Analysis (Day 0) | Wound Evolution Post-Grafting | Complete Healing |
|---|---|---|---|---|
| **Dog 4 (Group II)** | Injury progression for 6 months. | Clinically, an area with volume increase in Metatarsophalangeal region, proximal phalangeal region and right phalangeal region, firm consistency and without ulceration was observed. | After 48 hours, adherence of the scaffold to the tissue was observed, with the presence of a small amount of serous exudate. After 10 days, the wound showed a decrease in size, beginning with granulation tissue. | Total wound closure and fur growth after 45 days post-grafting. |

| | | | | |
|---|---|---|---|---|
| **Dog 5 (Group II)** | Complaint of ulcerative wound in the proximal portion of the left thoracic limb. Recurrent condition for at least 2 years. The histopathological result showed: Ulcerative dermatitis with areas of pyoderma and foci of fibrosis | Clinically, there was an ulcerated, erythematous lesion with discrete secretion in Lateral region of left forearm, large lesions and isolated ulcers in the proximal portion of the left thoracic limb. | 48 hours after grafting, a discrete presence of scaffold remnants was observed. On the eighth and tenth days after grafting, it was possible to observe regularization of the wound, discreet presence of exudate. In the twentieth, there was an onset of approximation of edges and peripheral healing evidenced by a whitish color on the edges. | Wound established 50 days after grafting. Total healing with the presence of fur. |
| **Dog 6 (Group II)** | Complaint of chronic injury in the distal portion of the left thoracic limb metacarpal region. | Clinically, a chronic lesion in Metacarpophalangeal region, proximal phalangeal region, proximal interphalangeal region | At 48 hours, partial presence of graft was observed, with regions with discrete exudate and slightly elevated surfaces. On the tenth day, the wound was partially closed, with a regular surface and fur growth. On | The complete healing process occurred at 50 days. |

| | | |
|---|---|---|
| | and left Middle phalangeal region, with hyperkeratosis, hyperpigmentation and edema was observed. | the twentieth day the adjacent scar tissue was prominent. |

**Supplementary Table S4.** Results indicating the patient's anamnesis and the macroscopic characteristics of the wounds before and after scaffolds grafting in dogs from group III (Control)

| Dogs | Historic | Wound Clinical Analysis (Day 0) | Wound Evolution Post Grafting | Complete healing |
|---|---|---|---|---|
| **Dog 7** **(Group III)** | Cutaneous nodule in the distal portion of the right thoracic limb (carpal region) with onset 1 year ago and progressive evolution. | A non-ulcerated nodular skin lesion in Right lateral metacarpal region was observed. | 10 days after grafting, there was a reduction in the diameter of the wound with the beginning of healing of edges and re-epithelialization. At 20 days, the wound was partially closed with exudate and the wound had an irregular edge. | Complete wound closure and partial fur growth occurred at 24 days. |

| | | | | |
|---|---|---|---|---|
| **Dog 8** **(Group III)** | Laceration by dog bite in the distal region of the left forelimb. | An open, deep and contaminated wound in Left plantar metacarpal region was observed, with purulent exudate. | 14 days after grafting, granulation tissue growth was observed with partial wound closure, serous exudate and irregular surface. 20 days after grafting, reepithelialization of edges and remodeling of the wound were observed. | Wound established 35 days after grafting, with discreet, thinning fur growth and presence of discreet serous exudate. |

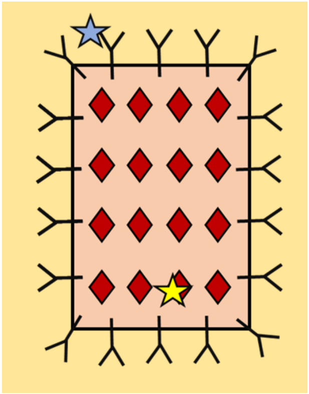

**Supplementary Figure S1.** Scheme of the surgical technique used for scaffolds grafting adapted from Paim [35] and Radlinsky [36]. Separate suture pattern, evidencing the distance between the stitches to bring the scaffold closer to the edges of the debrided wound (blue star). Scaffolds with staggered clefts (yellow star).
